# Supplementary material for: Competitiveness and sustainable development of Chinableapple industry
Source: PLoS One. 2022 Jun 1;17(6):e0268476. doi: 10.1371/journal.pone.0268476 (PMC9159607; doi:10.1371/journal.pone.0268476)
Supplement: Attachment — Submitted filename: Response to Reviewers.doc [file pone.0268476.s002.doc]

**Reviewer #1:**

**（1） The title of the article - is "Competitiveness and Sustainable Development of China’s Apple Industry". What does the author mean by the "Sustainable development" of the industry? The text of the papere is about the sustainable of competitiveness.**

Thank you very much for your valuable advice.This is explained in the introduction, lines 28-31. Sustainable competitiveness as understood in this paper is composed of some factors, and then it can bring about the sustainable development of apple industry. thank you The original text reads as follows:In the context of industry, sustainable competitiveness refers to the ability of an industry in one area, compared with the same type industry in other areas, to accrue and optimize resources and production factors to meet consumers’ needs and to survive and develop.

Thank you very much.

**（2）It is not clear from the text of the article why the author chose 2004 and 2018 for the study? And will the proposed methods of assessing competitiveness for other periods of time be relevant?**
Thank you very much for your valuable advice.This paper studies the sustainable competitiveness of apple industry. Firstly, the stability and representativeness of data research results should be ensured. Secondly, I consulted previous literatures and considered the research methods of previous scholars. Third, 2004 is the beginning of the study period, and 2018 is the end of a period. Therefore, this paper takes 2004 and 2018 as representative years to investigate the sustainable competitiveness of eight major apple producing areas.

**（3）I support the author's opinion that, of course, 8 regions are not enough to conduct a study (p. 19). Therefore, the evaluation index system of the apple industry competitiveness designed in this paper needs to be further improved.**

In the future, I will have the opportunity to study Apple industry in China through practical research. Considering the availability of data, the principle of representativeness and the experience of previous studies, eight major producing areas were selected as research regions. On the one hand, apple cultivation and production in China are mainly concentrated in these eight regions, so it is representative to select these regions for research. On the other hand, the data in China's agricultural statistics Yearbook and agricultural product Data compilation are only from these eight major apple producing areas, which are authoritative and reliable. thank you.

**Reviewer #2:**

Thank you very much for your valuable advice.

**1. Manuscript needs proofreading. There are a lot grammatical errors, e.g., line 147-148, line 153-155, product marketing refers….,..**
I recalibrated it. Thank you.

**2. The paragraph line 189 is very short.**
I rearranged it and put it at the end of the last paragraph.

**3. The author should justify why inverse distance weight matrix was used over the binary adjacency matrix or other methods**

I looked at the literature，the inverse distance weight method is a spatial distribution method that considers regional correlation among various factors and has been widely used in data processing due to its simple principle and accurate results. The inverse distance weighting matrix is based on the assumption that space effect intensity depends on distance. Except for the main apple-producing units, the effect size of all other evaluation units decreases with increasing distance, which is consistent with the first law of geography stating that "anything is always correlated, and things that are close are more correlated than things that are far away." Meanwhile, a non-sparse matrix can be transformed into a sparse matrix to solve the problem of long running time caused by excessive number of evaluations. Therefore, the inverse distance weight matrix is used as the basis for spatial autocorrelation analysis.

**4. Table 2 looks mixed up, the author should work on the table 2 and make it clear.**
The horizontal space of the table is limited, so I have changed the format according to the situation, please have a look.Thank you.

**5. Where is section 4.2? there are section 4.1 and section 4.3, please check.**
Section 4.2 is on page 9 of the paper.Thank you.

**6. The discussion should be elaborated by comparing the current study with the results from the previous studies.**
I changed the title of part 5 to discussion and policy implications, and compared the results of the current research with those of the previous research you said, added some literature and marked them in the paper.

**7. The conclusion should be brief and concise, there should be no numbering in the conclusion.**

According to your modification requirements, I have removed the serial number of the conclusion and deleted a few things.
